# Inhalation of a gastric polyp upon removal

**DOI:** 10.1055/a-2598-4735

**Published:** 2025-05-26

**Authors:** Ke Liu, Yuqing Chen, Xiaoli Ren, Lizhi Yi

**Affiliations:** 1 Department of Gastroenterology, The People’s Hospital of Leshan, Southwest Medical University, Leshan, China; 2 Department of Pathology, The Affiliated Hospital of Southwest Medical University, Luzhou, China

A 48-year-old man was admitted to our hospital for gastric polyp removal. His history and physical examination were unremarkable, and coagulation was normal.

After the patient had been anesthetized with propofol, esophagogastroduodenoscopy was performed and revealed a 2-cm type Isp polyp in the lesser curvature of the gastric fundus ( Fig. 1 **a, b** ). The polyp was removed by endoscopic mucosal resection. Thereafter, a snare was used to extract the sample. When passing through the esophageal entrance, the removed gastric polyp accidentally fell off the snare. When the gastroscope returned, the sample was inhaled into the trachea ( Fig. 1 **c** , Video 1 ). The patient was immediately placed in a semiprone position. Then, transnasal gastroscopy was performed with the intention of entering the trachea. Fortunately, before entering the trachea, the patient coughed out the specimen, which was eventually removed successfully ( Fig. 1 **d** ).

**Fig. 1:**
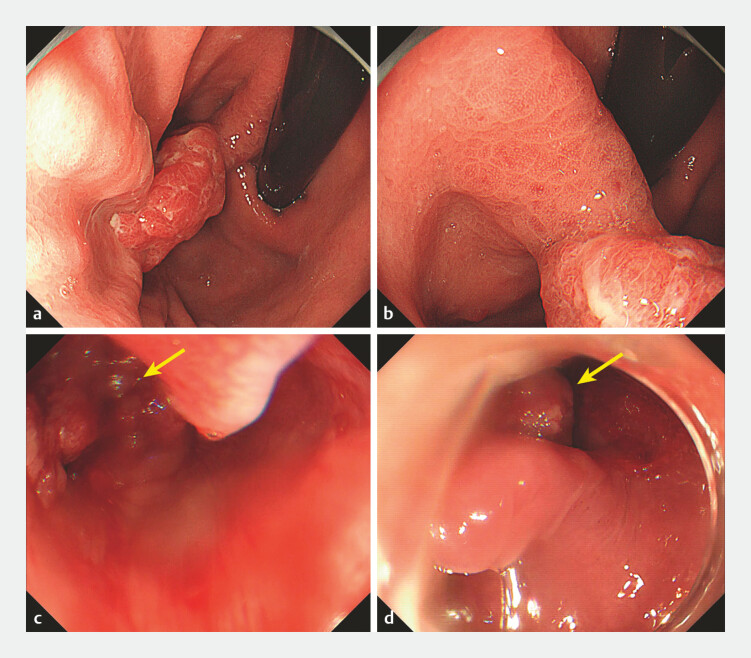
Endoscopy images. **a, b** Esophagogastroduodenoscopy revealed a 2-cm type Isp polyp in the lesser curvature of the gastric fundus. **c** The removed gastric polyp (arrow) fell into the hypopharynx. **d** The patient coughed out the specimen (arrow).

The specimen was inhaled into the trachea.Video 1

The patient experienced no discomfort postoperatively. Postoperative computed tomography of the lungs was normal. The histopathology of the specimen was gastric villous adenoma.

Common complications of gastric polyp resection include throat pain, abdominal distention, bleeding, and perforation 1 2 . Specimen aspiration has not been reported previously. It was inferred that the main cause of the aspiration was that the snare was pulled too loose. Our case shows that more attention should be given when the specimen passes through the entrance of the esophagus.

Endoscopy_UCTN_Code_CPL_1AH_2AZ

## Notes

Endoscopy E-Videos https://eref.thieme.de/e-videos
*E-Videos* is an open access online section of the journal *Endoscopy* , reporting on interesting cases and new techniques in gastroenterological endoscopy. All papers include a high-quality video and are published with a Creative Commons CC-BY license. Endoscopy E-Videos qualify for HINARI discounts and waivers and eligibility is automatically checked during the submission process. We grant 100% waivers to articles whose corresponding authors are based in Group A countries and 50% waivers to those who are based in Group B countries as classified by Research4Life (see: https://www.research4life.org/access/eligibility/ ). This section has its own submission website at https://mc.manuscriptcentral.com/e-videos .
